# Nandrolone Decanoate: Use, Abuse and Side Effects

**DOI:** 10.3390/medicina56110606

**Published:** 2020-11-11

**Authors:** Federico Giuseppe Patanè, Aldo Liberto, Andreana Nicoletta Maria Maglitto, Pasquale Malandrino, Massimiliano Esposito, Francesco Amico, Giuseppe Cocimano, Giuseppe Li Rosi, Dario Condorelli, Nunzio Di Nunno, Angelo Montana

**Affiliations:** 1 Legal Medicine, Department of Medical, Surgical and Advanced Technologies, “G.F. Ingrassia”, University of Catania, 95123 Catania, Italy; federicopatane90@gmail.com (F.G.P.); aldoliberto@gmail.com (A.L.); andreana.maglitto1992@gmail.com (A.N.M.M.); pasqualemalandrino@gmail.com (P.M.); massimiliano.esposito91@gmail.com (M.E.); francescoamico08@gmail.com (F.A.); peppecocimano@hotmail.it (G.C.); dariocondo@hotmail.it (D.C.); 2 Department of Law, Criminology, Magna Graecia University of Catanzaro, 88100 Catanzaro, Italy; lirosigiose@gmail.com; 3 Department of History, Society and Studies on Humanity, University of Salento, 73100 Lecce, Italy; nunzio.dinunno@icloud.com

**Keywords:** nandrolone decanoate, drug abuse, androgenic steroids, adverse effects, steroid treatment

## Abstract

*Background and Objectives:* Androgens play a significant role in the development of male reproductive organs. The clinical use of synthetic testosterone derivatives, such as nandrolone, is focused on maximizing the anabolic effects and minimizing the androgenic ones. Class II anabolic androgenic steroids (AAS), including nandrolone, are rapidly becoming a widespread group of drugs used both clinically and illicitly. The illicit use of AAS is diffused among adolescent and bodybuilders because of their anabolic proprieties and their capacity to increase tolerance to exercise. This systematic review aims to focus on side effects related to illicit AAS abuse, evaluating the scientific literature in order to underline the most frequent side effects on AAS abusers’ bodies. *Materials and Methods:* A systematic review of the scientific literature was performed using the PubMed database and the keywords “nandrolone decanoate”. The inclusion criteria for articles or abstracts were English language and the presence of the following words: “abuse” or “adverse effects”. After applying the exclusion and inclusion criteria, from a total of 766 articles, only 148 were considered eligible for the study. *Results:* The most reported adverse effects (found in more than 5% of the studies) were endocrine effects (18 studies, 42%), such as virilization, gynecomastia, hormonal disorders, dyslipidemia, genital alterations, and infertility; cardiovascular dysfunctions (six studies, 14%) such as vascular damage, coagulation disorders, and arteriosus hypertension; skin disorders (five studies, 12%) such as pricking, acne, and skin spots; psychiatric and mood disorders (four studies, 9%) such as aggressiveness, sleep disorders and anxiety; musculoskeletal disorders (two studies, 5%), excretory disorders (two studies, 5%), and gastrointestinal disorders (two studies, 5%). *Conclusions:* Based on the result of our study, the most common adverse effects secondary to the abuse of nandrolone decanoate (ND) involve the endocrine, cardiovascular, skin, and psychiatric systems. These data could prove useful to healthcare professionals in both sports and clinical settings.

## 1. Introduction

The name “anabolic androgenic steroids” already suggests their “anabolic” (from Greek ἀναβολή “throw upward”) and “androgenic” (Greek ἀνδρός “of a man” + -γενής “born”) properties.

Androgens play a significant role in the development of male reproductive organs, such as the prostate, penis, seminal vesicle, ductus deferens, and epididymis. Testosterone is a steroid hormone that has an essential role in the development of the male phenotype and the regulation of reproduction of males. This hormone is effective on puberty, fertility, and sexual function in males [1,2].

Anabolic androgenic steroids (AAS) represent a large group of synthetic derivatives of testosterone, produced to maximize anabolic effects and minimize the androgenic ones [3]. Several structural modifications have been introduced into testosterone in an attempt to maximize the anabolic effect and minimize androgenic effects. Currently, AASs are classified in 3 major classes [4] based on substitution of the base molecule. Class I is related to C-17 esterification. Class II is related to a demethylated group at C-19 and may also have C-17 esters. Class III is related to alkylation at C-17.

The classification of anabolic steroids is given in Table 1.

Nandrolone is included in the group of class II AASs, which is composed of 19-nortestosterone-derivates. In general, AASs is a broad and rapidly growing group of synthetic androgens used both clinically and illicitly.

Compared to testosterone propionate, nandrolone decanoate is considered to have strong anabolic effects but weak androgenic effects (potency ratios of 3.29–4.92 and 0.31–0.41). In particular, nandrolone esters are thought to have the highest ratio of anabolic to androgenic effects of any AAS. The low androgenicity of nandrolone decanoate is thought to be due to the fact that nandrolone is inactivated by 5α-reductase via transformation into the low-affinity androgen receptor (AR) ligand 5α-dihydronandrolone. This is thought to result in a lower incidence and magnitude of side effects.

Nandrolone has very low affinity for human serum sex hormone-binding globulin (SHBG), about 5% of that of testosterone and 1% of that of dihydrotestosterone (DHT). It is mainly metabolized by the enzyme 5α-reductase, into 5α-dihydronandrolone, 19-norandrosterone, and 19-noretiocholanolone, which can be detected in urine [5]. Nandrolone displays a so-called flip-flop pharmacokinetics. This means that the ascending phase of the curve represents the disposition of nandrolone, and the descending part of the curve represents the rate-limiting process of release of nandrolone decanoate from the muscle into the general circulation [6]. In clinical use, nandrolone is applicable in clinical practice for burns, radiation therapy, surgery, trauma, and various forms of anemia [7]. Moreover, it has also been used for the treatment of chronic kidney disease, osteoporosis in postmenopausal women [8], inoperable breast cancer, and for patients on long-term corticosteroid therapy, as well as an adjunct to therapy for conditions characterized by a negative nitrogen balance. The drug is often used off-label to preserve lean mass in human immunodeficiency virus (HIV)/acquired immunodeficiency syndrome (AIDS) associated wasting syndrome [9].

The compound is famous not only among adults, but also adolescents because of its anabolic, muscle-building properties [10,11,12,13]. Skeletal muscle can be considered as the primary target tissue for the anabolic effects of AAS, which are mediated by androgenic receptors which, after exposure to AAS, are up-regulated, and their number increases with bodybuilding [14]. Therefore, AAS determine an increase in muscle size as a consequence of dose-dependent hypertrophy resulting in an increase of the cross-sectional areas of both type I and type II muscle fibers and myonuclear domains [15]. It is administered via intramuscular injection and is metabolized in a similar manner to testosterone, with conversion into 3-norandrosterone by5α-reductase [16]. The recommended therapeutic dose of ND for humans is 0.4 mg/kg/day [17]. Its consumption can trigger a series of adverse side effects in the body, both acute and chronic [18]. However, acute adverse effects have also been described, primarily consisting of headaches, fluid retention, gastrointestinal irritation, diarrhea, abdominal pain, jaundice, menstrual abnormalities, and hypertension. The chronic effects of AAS abuse, aside from neuropsychiatric and behavioral effects, include a wide range of somatic consequences. Many organs and systems are targets of AAS action. Consequently, AASs may exert negative effects on reproductive, hepatic, musculoskeletal, endocrine, renal, immunologic, cardiovascular, cerebrovascular, and hematological systems [19,20,21,22].

Moreover, it has been reported that AASs can increase tolerance to exercise by making the muscles more capable of resisting overload, thereby shielding them from muscle fiber damage and improving the level of protein synthesis during recovery [23]. 

Nowadays, especially athletes in power sports such as bodybuilding and weightlifting administer illegally high doses of AASs to increase their muscle mass and improve their overall performance [24]. However, also non-athletes also abuse AASs. Nandrolone decanoate (ND) injection has been classified as a Schedule III controlled substance under the Anabolic Steroids Control Act of 1990 [25]. Due to serious health risks, the nonmedical use of AASs is banned by most sports organizations. In addition, AASs are listed in the WADA (World Anti-Doping Agency) prohibited list [26]. The abuse of these drugs has become a major health problem [27].

The use of AASs in competitive bodybuilding became widespread and was often supervised by physicians who supplied the drugs to the athletes, ensuring what they were injecting was pure while monitoring and minimizing side effects such as infertility, liver toxicity, impaired lipid profiles, high blood pressure, acne, hair loss or gynecomastia. During this time, there was no need for a black market or underground laboratories (UGL) since these drugs were readily available from health professionals. However, the situation dramatically changed after the introduction of the Anabolic Steroid Control Act in 1990, and subsequently reinforced by the Anabolic Steroid Control Act of 2004. In 2014, the Designer Steroid Control Act was enacted in an attempt to close loopholes for slightly modified compounds. These events created an immense demand for black market products, which facilitated the creation of underground laboratory products and importing drugs produced in countries with lax AAS legislations [28].

Even though legitimate pharmaceutical grade AASs can be purchased on the black market via several routes, physician supervision of usage is usually lacking, making a legitimate pharmaceutical product potentially dangerous for uninformed users. Since buying and using AASs (without a medical prescription) is a criminal act in many countries, the AAS user is often reluctant to seek advice from a physician when health issues arise. Indeed, a survey found that AAS users very often have no trust in physicians’ knowledge about AASs and typically do not disclose their AAS use to them [29]. 

The aim of this systematic review is to focus on the side effects related to illicit AAS abuse, evaluating findings in the scientific literature, in order to underline the most frequent side effects on AAS abuser’s bodies.

## 2. Materials and Methods

### 2.1. Database Source

We performed a systematic review of the literature on online resources using the PubMed database for all published articles from 1 January 1900 to 22 July 2020, using the key words: “nandrolone decanoate”.

### 2.2. Selection of Studies and Data Collection

**Figure 1:**
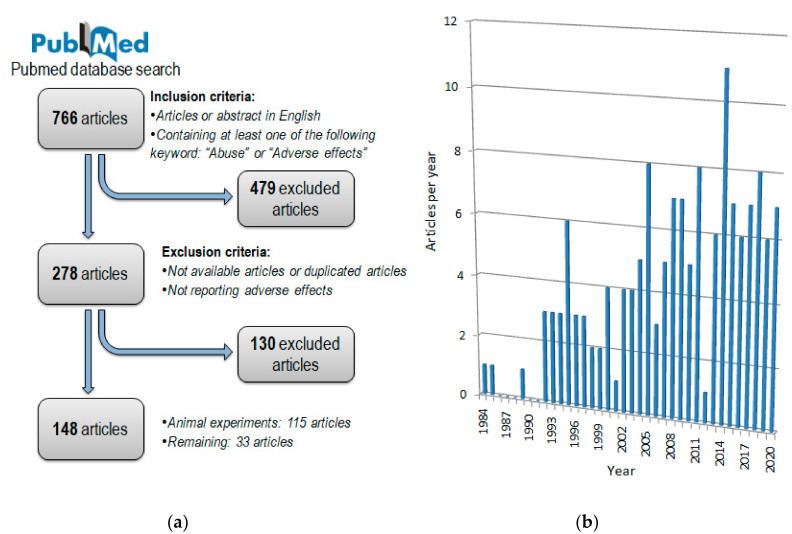
Included articles search strategy; (**a**) The flowchart about article selection used for the literature review. (**b**) Time distribution of included articles: *x*-axis for year; *y*-axis for amount of articles per year.

A total of 766 articles were retrieved, excluding all duplicated articles, additional exclusion and inclusion criteria were then applied: Articles or abstracts in English containing one of the following words: “Abuse” or “Adverse effects”. A total of 479 articles did not meet these criteria and were therefore excluded. From the remaining 278 articles a manual review was performed to remove non-available articles, duplicate articles, articles not relevant for the study, older literature reviews and articles not reporting adverse effects. A total of 148 articles met these inclusion criteria and were considered eligible for the study, while 130 articles were excluded. Studies from the references of the selected articles, and articles not meeting inclusion and exclusion criteria are discussed in this study, but not included in the systematic review. For a more comprehensive review, we included in the discussion the excluded but relevant articles, eventually performing a specific research for key sections. Figure 1 summarizes the flowchart about article selection after the PubMed search. All included articles are listed in Appendix A.

## 3. Results

A total of 115 studies reported data about animal experiments (for example rats, horses, dogs), while 33 studies reported data about humans (409 subjects, 346 males, 63 females) as shown in Table 2. The subjects mean age was 34.8 years (Standard Deviation: 12.8), as described in 21 articles. Males were younger (33.3 years) than females (53.3). Eighteen articles discussed side effects of subjects abusing nandrolone decanoate (without medical prescription), while 15 articles discussed side effects of subjects taking it for medical treatment.

**Figure 2:**
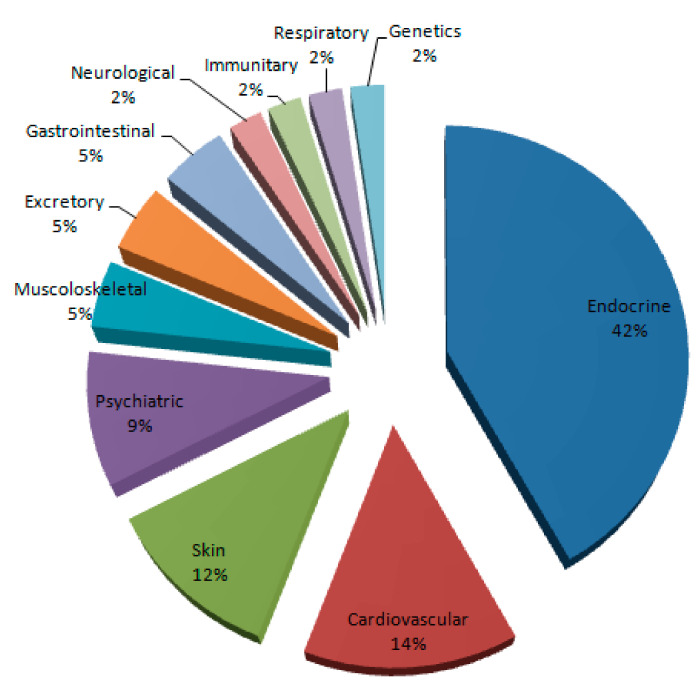
This graph summarizes the adverse effects reported. Endocrine, cardiovascular, skin and psychiatric disorders are the most reported.

Adverse effects were studied on these population-based studies. The results are shown in Table 3 and Figure 2. Most reported adverse effects (more than 5% of the studies) were endocrine (18 studies, 42%), cardiovascular (six studies, 14%), skin (five studies, 12%), psychiatric (four studies, 9%), musculoskeletal (two studies, 5%), excretory (two studies, 5%), and gastrointestinal disorders (two studies, 5%). In order to list all adverse effects, these were grouped by affected system based on study reports (for example, in psychiatric disorders, we grouped all behavioral, mood, and anxiety symptoms).

The most common side effects were: endocrine disorders (virilization, gynecomastia, hormonal disorders, cholesterol and lipid disorders, genital and infertility issues); cardiovascular disorders (vascular damage, coagulation disorders, arteriosus hypertension); skin disorders (pricking, acne, skin spots); psychiatric disorders (aggressiveness, mood disorders, sleep disorders, anxiety); musculoskeletal disorders (tendon ruptures); excretory disorders (organ damage); gastrointestinal disorders (organ damage and liver adenomas); neurological disorders (seizures); immune disorders (chronic infection relapse; respiratory disorders (sleep apnea syndrome); genetic disorders (genetic damage).

**Figure 3:**
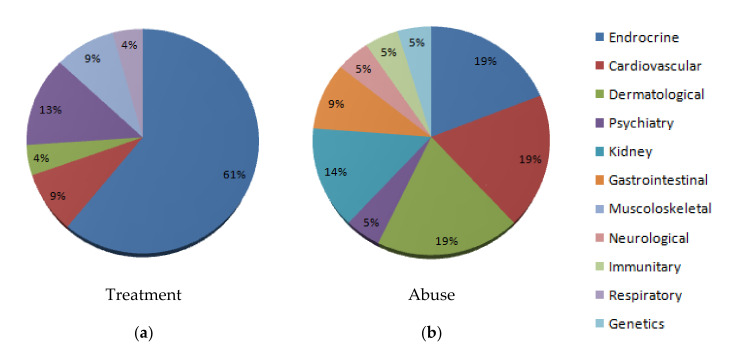
Graph representing side effects for treatment or abuse related administration. (**a**) Treatment related. (**b**) Abuse related.

**Figure 4:**
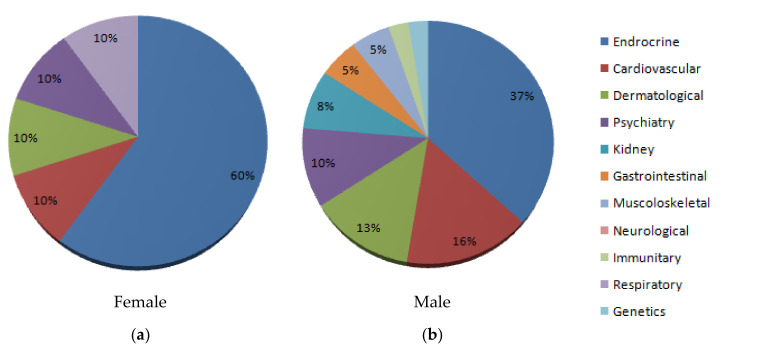
Graph representing side effects for males and females. (**a**) Females. (**b**) Males.

We also examined the difference between reported side effects between men and women, as well as between the medical administration and abusive use. The most reported side effect concerns the endocrine system for both males and females; reported more frequently for females. The most reported side effects for treatment administration were on the endocrine system (61%), while the most adverse effects for abusive use were endocrine (19%), cardiovascular (19%), and dermatological disorders (19%). Reports related to males were mixed between treatment and abusive use (45% were from reports of medical treatment, while 55% were from reports of illicit or recreational use). In our data, the most common reported side effect was the endocrine system for both males and females (60% for females, 37% for males). We think this result is caused by the number of reports: most examined female cases were secondary to treatment side effects, while male cases were related to both treatment and abuse, as shown in Figure 3 and Figure 4.

Moreover, the incidence of side effects on males was more homogeneous (37% endocrine disorders, 16% cardiovascular disorders, 13% skin disorders, 10% psychiatric disorders, compared to females where we found 61% endocrine disorders, 10% cardiovascular disorders, 10% skin disorders, 10% psychiatric disorders). We think this difference is due to the administration dosage and pattern. In fact, most abusers use several AAS at the same time.

## 4. Discussion

AASs represent a large group of synthetic derivatives of testosterone designed to maximize anabolic effects (such as increasing muscle mass and strength and reducing fat) and to minimize androgenic ones (such as virilization) [30,31,32]. There are numerous clinically proven effects: AASs promote stimulation of growth and maturation of non-reproductive tissues and maintenance of secondary sexual characteristics and reproductive function [33]. They also promote bone growth in both young and adult populations; and have long been used as a treatment for growth delay and osteoporosis [30,34]. Anabolic androgenic steroids are clinically indicated for the treatment of chronic diseases associated with the catabolic state of the patient, in conditions of AIDS, chronic obstructive pulmonary disease, hepatic or renal failure, cancer, and in cases of burns and postsurgical recovery. They are also recommended for androgen replacement therapy after menopause, and during age-related sarcopenia [35,36].

ND is the most prescribed AAS because it exhibits the lowest incidence of adverse effects compared to beneficial effects [37]. As a result, ND had been implicated in doping, until its health risks became evident and the International Olympic Committee (IOC) abolished the use of ND in sports competitions [38]. While it is more common among so-called gym visitors, this substance is also used in criminal circles and in competitive situations where personal aggressiveness could be a determining factor.

ND is generally used in the injectable form to improve performance [23], regularly or occasionally, with a combination of multiple AASs. Athletes tend to self-administer AASs for several weeks before sports competitions believing in the synergic anabolic effects with minimal side effects and the possibility of avoiding being discovered on doping tests. [39,40].

**Figure 5:**
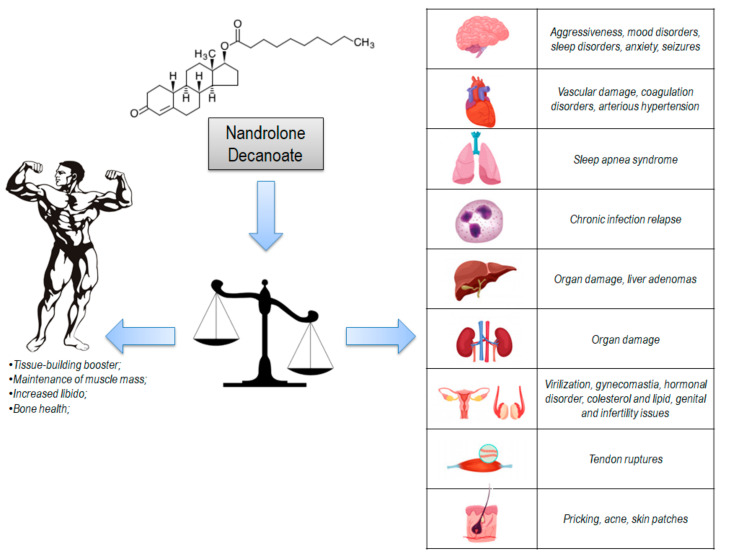
Where is the balance between known positive effects and underestimated or unknown side effects? This image tries to summarize this concept, because nandrolone decanoate is a molecule that affects several systems at the same time and sometimes in an irreversible way.

The anabolic effect on proteins requires a specific diet necessary to maintain a correct nitrogen balance. Unfortunately, hyperproteic diets are often not balanced and excessive proteins are eliminated through urine or converted into fat [39,41]. A summary of these effects is represented in Figure 5.

In order to investigate the side effects of such abusive administration of ND, we reviewed the literature and studied the results systematically.

### 4.1. Endocrine and Genital Disorders

In our data, the most reported endocrine disorders were serum lipid alteration and virilization (for example, gynecomastia, voice pitch alteration). Several authors, after a period of administration of AASs, highlighted a significant increase of low-density lipoprotein (LDL) and decreasing high-density lipoprotein (HDL). Such effects and reversibility are dependent on dosage and treatment duration [30,42,43]. It has been shown that high doses of AASs induce adverse effects by increasing plasma triglyceride levels and decreasing plasma HDL-C levels up to 70%, considered to provide anti-atherosclerotic protection [44], while some studies showed contradictory results about this aspect [45,46].

ND has important effects on the hypothalamic—pituitary—adrenal Axis (HPAA) and on lipid metabolism. In a study by Gårevik et al., cholesterol level, steroid synthesizing enzymes in the adrenal gland (HMGCR) mRNA and Apo-lipoprotein B (ApoB) appeared to increase after a single dose of ND in humans, and this effect was persistent after 14 days [47]. Belgoma and colleagues [48] highlighted the molecular mechanism behind serum lipid alteration, with a downregulation of several intracellular factors that leads to the synthesis of sphingolipids and glycerolipids. AASs decrease lipogenesis by the downregulation of the activity of the lipogenic liver X receptor pathway via activation of the androgen receptor. Moreover, the androgen receptor induces de novo synthesis of fatty acids and cholesterol by upregulated HMG-CoA (steroid synthesizing enzymes in the adrenal gland—Coenzyme A) reductase and low-density lipoprotein (LDL) receptor.

Racca and colleagues [49], after having administered ND to rats, noted an increase in adrenocorticotrophin (ACTH) (both in blood and in pituitary corticotropes), glucocorticoid receptor (GR) reduction in the hippocampus and hypothalamus cytosol, and GR translocation in the hippocampus nuclear fraction, stimulation of cortical serotonin re-uptake and activation of hippocampus cytosolic extracellular signal-regulated kinases 2 (ERK2). Alsio and coworkers [50] noted an important reduction in corticosterone (CORT) plasma levels in the rat after ND treatment for 14 days; Nandrolone treatment increased HMGCR expression in the adrenal glands and reduced expression levels of the b3-adrenoceptor in adipose tissue [51].

Cases of women with ovarian follicles maturation failure, uterus, clitoris, vagina, and mammary gland hypertrophy, with hormonal disorders, confirmed by experiments on animals, have been reported in the literature. ND decreased the serum level of follicle-stimulating hormone (FSH), Luteinizing hormone (LH), progesterone and estrogen [52]. ND also promoted histological alterations in female genital organs in a dose-independent manner, despite recovery from treatment [33,53].

Virilization in female users is a well-known side effect, characterized by voice pitch alteration, body hair growth, hair loss, thick and greasy skin, acne, as well as increased libido and clitoris hypertrophy [54].

AASs also affect the activity of the sexual hormone in males, causing the inhibition of reproductive function, since the first administration [47], by turning off physiological testosterone production, causing testis atrophy, spermatogenesis inhibition (also leading to aspermia), and erectile dysfunction. They also cause biochemical modifications of prostatic secretion and seminal liquid. Most of these degenerative changes are partially reversible after treatment suspension [33,55,56]. 

ND, among other AASs, exerts a strong negative feedback on the hypothalamic-pituitary-gonadal axis that reduces the levels of LH and FSH and leads to a reduction of testosterone. Due to the decrease in FSH levels, the growth and development of Sertoli cells is insufficient [56].

### 4.2. Cardiovascular Disorders

The effects of androgens on the cardiovascular system involve blood vessel disorders, increased erythropoiesis, hematocrit increase, hyperviscosity and hypertension, but may have direct effects on cardiac muscle and its function, decreasing potential duration, altering repolarization, and peak shortening times [57,58].

In our data, the most reported cardiovascular disorders were platelet aggregation disorders and cardiac injuries. Shirpoor and coauthors [59], through experiments on rats, showed the molecular mechanisms underlying heart hypertrophy: chronic nandrolone treatment with or without strenuous exercise causes a shift in the alpha and beta–myosin heavy chain (α-MHC/β-MHC) isoform expression manifested by elevation of β-MHC mRNA and the ratio of β-MHC mRNA/α-MHC mRNA expression, as well as an increase in the heart tissue of mono-amine oxidase (MAO) and calcium/calmodulin-dependent protein kinase II-δ activities (CaMKII-δ). Moreover, androgens have receptors in the heart and their action directly affects it through coupling directly with nuclear receptors and increasing the expression of mRNA, thus stimulating cardiac protein synthesis resulting in myocardial hypertrophy [60,61].

Franquni and coauthors [62] showed the presence of cardiac remodeling and subsequent cardiac injury as indicated by the reduction in cardiac troponin I. Additionally, a reduction in the Bezold–Jarisch reflex (BJR) control of heart rate (HR) and blood pressure was also demonstrated. As a result of these changes, animals treated with ND demonstrated increased blood pressure that reached hypertensive levels. These mechanisms are related to the ability of ND to produce a reduction of the anti-inflammatory cytokine (IL-10) and augmentation of the pro-inflammatory cytokines (IL-6 and TNF-a) causing cardiac remodeling and injury [63,64,65,66].

In a study by Omar and colleagues, the authors suggested a mechanism of androgenic stimulation of platelet aggregation through either increased production of thromboxane A2 or decreased prostacyclin and cyclooxygenase activity, synergistic with polycythemia, and increased platelet count thus causing increased blood viscosity [67]. Pro-aggregatory effects on platelets because of high dosages of androgens could be related to a decrease in cycloxygenase activity [68]. Most of these alterations could lead to an increased thrombosis risk or atherosclerotic effects on vessels [42,69].

Many studies have tried to identify a direct relationship with heart disorders by performing animal experiments and identifying autonomic dysfunction [70,71], fibrosis, hypertrophy, and myopathy [56,72,73], affecting ionic balance across the organ with a probable synergistic effect with other drugs [74].

Almaiman and colleagues showed an increase in creatine kinase (CKL) and creatine kinase muscle and brain subunits (CK-MB) [30]. In a case reported by Huie and coworkers, an acute myocardial infarction in an anabolic steroid user, raised questions about any link between them [75]. 

Most AAS abusers tend to use multiple substances at once, causing synergic effects and systemic disorders whose causes cannot be quickly identified by physicians. In a study by Clark and colleagues, the authors reported a case of a subject using several drugs, thus causing dilated cardiomyopathy [76].

Several molecular pathways are implied in androgen-induced cardiac damage, where nuclear and cytoplasmatic factors play a role [77]. ND abnormally affects ionic balance in several ways, including altered Ca^2+^ mobilization [74] downregulated K^+^ channel-interacting proteins causing longer QT repolarization time [78], along with increased oxidative stress and pro-apoptotic effects [79].

Although previous studies verified the association between AAS exposure and high blood pressure, the molecular mechanisms involved in blood pressure increase due to AASs are not fully understood. Some studies, however, have suggested that mechanisms such as changes in sodium balance, degenerative vascular lesions, cardiac hypertrophy, and an unfavorable lipid profile exist [80,81]. Franquni M. and colleagues showed that myocyte hypertrophy and cardiac remodeling (ND related) are related to augmentation of ACE activity and the development of a pro-inflammatory state, and consequent cardiac changes result in the development of hypertension in animals treated with ND [62].

### 4.3. Skin Disorders

In our data, the most reported skin lesions were colored patches, acne, and itch disorders. Almaiman and colleagues, in a study conducted on a group of gym athletes who were using a mix of several AASs, reported itching and the emergence of skin patches among other adverse reactions [30]. Similar skin characteristics were highlighted in another case report by Tripathi and colleagues, of a 55-year-old woman [82]. Self-injection of ND in external genitalia was reported as the cause of a paraffinoma and an above skin ulceration by Balighia and colleagues, in a 56-year-old man [83]. Such findings are in line with previous literature findings [19].

### 4.4. Psychiatric and Neurological Disorders

Evidence indicates the potential role of AASs in modifying behavior with symptoms such as anxiety [84], concentration defects, irritability, and even violence during a long-term administration. In contrast, when the administration was stopped, the reported side effects were melancholy and depression [82,85,86].

Aggressiveness is a common finding in AAS abusers, among other psychiatric disorders [87], and this has been confirmed by several studies on animals [88,89,90,91,92,93].

Several studies on animals confirmed neurotoxic effects of AASs in the brain. Turillazzi and colleagues demonstrated the role played by oxidative stress, thus causing an apoptotic response in the rat brain after chronic treatment with nandrolone decanoate [94]. Chronic exposure permanently influences the expression of serotonergic and noradrenergic neurotransmission [95].

Both human and animal studies have shown dysfunction of visual-spatial memory after AAS use. Magnusson and colleagues propose that administration of nandrolone to male rats may affect memory function via dynorphinergic actions [96,97].

Seitz and colleagues highlighted an increased amygdala volume and reduced resting-state functional magnetic resonance imaging (MRI) coupling of the amygdala with cognitive control and memory regions in AAS abusers. The authors concluded that long-term AAS use might alter amygdala-related functional and structural brain networks [85,98].

Moreover, Selakovic and colleagues suggested the possibility that alterations in hippocampal parvalbumin interneurons (i.e., GABAergic system) may be involved in anxiousness induced by ND abuse [99].

Chronic treatment with ND has been associated with impact on both opioid concentrations and tachykinin levels in brain areas connected with the control of emotional behavior such as depression, aggression, and reward.

The androgen action is related to its ability to bind and activate AR. The immunoreactivity of substance P (SP), which is a peptidergic factor associated with enhanced aggression in several brain regions, namely the amygdala, hypothalamus, periaqueductal gray area, and striatum [100], has been shown to increase after ND administration. ND has also been shown to react on the Substance P system at several levels, including receptor densities, peptide concentrations, and enzymatic processing [101,102]. 

ND may induce its effect directly through AR, causing oxidative stress and different effects across the brain [103]. Moreover, serotonin, glutamate, and dopamine systems, activation of gamma-aminobutyric acid (GABA) and N-methyl-D-aspartate (NMDA) receptors as well as the activation of steroid receptors, such as estrogen, mineralocorticoid, progesterone, and glucocorticoid receptors, could all contribute to the altered behaviors described. Increased aggressive behavior has been shown in many studies but there is no univocal opinion of authors because of different methodological approaches [90]. While several studies correlate severity and duration of symptoms with chronic ND administration, it is already known that a single injection of ND is enough to alter brain activity: a hyper-adrenergic state with an increased amount of 5-hydroxytryptamine (5-HT) metabolites in the hypothalamus, after a single dose [17], altered the reward system by affecting dopamine metabolism [104], and altered monoamine metabolism [105].

### 4.5. Musculoskeletal Disorders

In our collected data, we identified only two studies that reported adverse effects of ND, both concerning abusers.

Liow and colleagues reported a case of a 29-year-old male who abused a mix of several AASs and got a bilateral rupture of the quadriceps tendons [106], while Stennard and colleagues presented a case of isolated rupture of the triceps tendon in an athlete who was lifting weights [107]. Both cases suggest that oral steroid abuse may cause negative effects on the mechanical properties of connective tissue, confirming the experimental study of Marqueti and colleagues [108]. In animal experiments, anabolic steroids produced a stiffer tendon that absorbs less energy and fails with less elongation [109].

The molecular mechanism underlying this altered tendon activity may be related to collagen synthesis [60,110]. Hassager and colleagues concluded that anabolic steroids stimulate type III collagen synthesis, which affects muscular tissues as well as bone tissues [111].

### 4.6. Excretory and Liver Disorders

Hepatotoxicity is one of the most frequent side effects of AAS abuse [112,113]. AAS-induced hepatotoxicity has been hypothesized to be related to oxidative stress in hepatic cells. Indeed, because of AR activation an increase in reactive oxygen species can be observed due to the increase in mitochondrial b-oxidation. Moreover, antioxidant substances have a protective role against hepatotoxicity mediated by AASs. It has also been demonstrated that androgenic potency and metabolic resistance are positively linked to the degree of liver damage.

AAS-induced hepatotoxicity is influenced by genetic factors and is related to the infiltration of inflammatory cells in liver tissue, such as lymphocytes, neutrophils, and eosinophils. Oxidative stress could play a role in determining liver damage consequently to AAS abuse by activating androgen receptors that lead to mitochondrial degeneration of hepatic cells. A recent study evaluated the liver effects of five weeks of ND administration in rats. The results highlighted an increase of plasma levels of liver necrosis markers, an increase in collagen deposition in liver parenchyma, portal space, and centro lobular vein [113,114]. The mechanism involved in collagen deposition could be the increase in the number and in the activity of Kuppfer cells. In this regard, Kuppfer cell activation leads to the production of many inflammatory cytokines, such as transforming growth factor beta 1 (TGF-b1), nuclear factor kappa-light-chain-enhancer of activated B cells (NF-Kb), and interleukin 1 beta (IL-1 β), related to the liver fibrosis process [115,116]. 

Many AASs can be administrated in parenteral or oral ways, causing different metabolism altering androgenic or anabolic effects. ND is injected intramuscularly with an oil that delays absorption and is not hepatotoxic [16,117].

Several studies highlighted that prolonged androgen exposure has a direct toxic effect on kidneys, especially glomerular cells, causing the accumulation of mesangial matrix, podocyte depletion and structural adaptations [118,119]. In this regard, kidney tissues are characterized by the expression of ARs. AR activation leads to cell growth and hypertrophy in the kidney. A recent report suggested that ND exposure promotes hypertrophy in proximal and distal convoluted tubules of mice kidneys [120]. Moreover, both testosterone activity and ND direct action to AR may play a role in the genesis of kidney fibrosis after long-term ND exposure [121]. 

Prolonged ND administration in mice has been shown to cause dose-dependent oxidative kidney stress and damage. Indeed, mice kidneys treated with ND exhibited increased lipid peroxidation and decreased antioxidant enzymes activity, such as glutathione reductase and glutathione peroxidase. A recent study suggested a dose related oxidative stress in mice kidneys treated with prolonged doses of ND [118]. The authors observed an increase in markers of lipid peroxidation and an increase of pro-inflammatory and pro-apoptotic markers, such as IL-1 β, heat shock protein 90 (Hsp90), and tumor necrosis factor (TNF) associated with a decrease of antioxidant enzymes, which could lead to secondary focal segmental glomeruloscelerosis [118].

Bagchus and colleagues, studied healthy men after injecting ND: urinary metabolites were detectable for at least 33 days after injection and the serum concentration of ND showed a half-life of 7–12 days [38].

Increasing bilirubin, alkaline phosphatase, and transaminases are the most frequent evidence in blood. Several studies evidence the role of ND in functional and morphological liver and kidney changes, thus developing an increase of creatinine, urea, alanine transaminase and aspartate transaminase blood levels [30,122].

Kidney and liver histological changes in ND users are usually fibrosis and cell proliferation. The causes of this degenerative process are multifactorial, but much evidence shows that oxidative stress is involved [19,60,110,111,118]. During ultrasound examination, kidneys usually show increased volume and cortical thickness in bodybuilders who regularly take anabolic steroids. These findings are in line with the theory of a multifactorial association of steroid, hyperproteic diets and intensive sport training being involved in renal damage [94,123] along with hypertension and fluid retention that could probably be associated with a decreased level of kidney α1B-adrenoceptors [124,125].

Long administration could cause hepatic peliosis, fibrosis and hepatic cancer [126], and related alteration of cellular redox balance [111,115]. Wen-Lung and colleagues studied the role of AR on different liver diseases, but univocal results have not yet been obtained. Moreover, the underlying molecular mechanism is not well defined [127].

### 4.7. Immune Disorders

In our collected data, we identified only one case concerning adverse effects of ND on abusers, reported by Singh and colleagues, namely a 21-year-old man who started with a mix of anabolic steroids, with the emergence of a rare serious adverse effect of suspected tubercular reactivation [46,128].

Supra-physiologic doses of common AASs alter immune function by influencing the production of certain cytokines. In fact, the users of AASs have abnormal immunoglobulin (Ig) concentrations; the lowest levels of immunoglobulin G (IgG), immunoglobulin M (IgM) and immunoglobulin A (IgA), “significantly lower” than controls for IgA and IgM [129]. Kanda and coauthors [130] showed that spontaneous IgM and IgG production in humans was inhibited by exposure to 1 nM testosterone, which is nearly a physiological dose, suggesting that high doses would potentially adversely affect the immune system. Some studies suggest that AASs are immune suppressive and depend on the type of AAS used and the dose and timing of administration.

AASs increase serum hemoglobin concentrations, improving the aerobic capacity in athletes: two studies recorded an AAS-induced alteration of hematology in athletes [131,132]. Alen demonstrated an increase in serum hemoglobin concentration and hematocrit, platelets, and white blood cell count after six months of high dose AASs. Similarly, Hartgens and coworkers found an increase in platelet count after short-term dosing (eight weeks) of AASs. The significance of these studies is that they indicate that AAS abuse can potentially affect erythropoiesis and other hematological parameters.

Hughes and associates [133] determined that supraphysiologic doses of nandrolone decanoate and oxymethenelone enhanced the production of the inflammatory cytokines IL-1β and TNF-α in human peripheral blood lymphocyte cultures in vitro.

On this basis, it can be hypothesized that the chronic administration of nandrolone, favoring the persistence and viability of stem cells in different tissues, could represent a preconditioning that, in addition to multiple hits, could enhance the risk of carcinogenesis onset especially in stem-cell-rich tissues such as liver [40]. The side effects on the natural synthesis of anabolic steroids play a potential role on hormonal changes/regulation and they could be suspected to be at the base of certain carcinogenic mechanisms [134,135]. Furthermore, easily accessible and commonly diffused AASs, such as nandrolone and stanozolol, playa potential role in the pathogenesis of cancer, such as Leydig cell tumor, through multiple process pathways [134]. Nandrolone magnified cyclin D1 concentration, inducing breast cell proliferation. These processes, individually or in combination, can induce micronuclei formation that are strictly related to several mutagenic stresses and are formed following chromosomal damage eliciting profound modifications in genetic sequences by means of alterations in telomerase activity [136].

Nandrolone is an androgen receptor agonist. On binding to the AR, it may induce the release of the AR receptor from Hsp90 and its translocation to the nucleus; higher nandrolone concentrations induced a more pronounced increase in Hsp90 levels of expression and phosphorylation. This result is an indirect demonstration that nandrolone binds to AR and induces its activation. Hsp 90 was found to be overexpressed in multiple cancers, including prostate cancer [137].

## 5. Conclusions

The anabolic-androgenic steroids are a family of hormones abused by athletes because of their well-known properties on increasing muscle mass and strength, and among them ND is the most used one. Historically, it was used for the treatment of anemia of chronic kidney disease, or osteoporosis in postmenopausal women.

This review evidences that improper usage and abuse of AASs cause several adverse effects in all body tissues and organs, highlighting the mechanics behind side effects. To sum up, inflammatory cytokines, oxidative stress, protein synthesis alteration, and apoptosis are common mechanisms involved in AAS-related damage.

Several studies showed cardiovascular and endocrine system, reproductive system, musculoskeletal system, as well as kidney and liver are affected by side effects in most cases. To date most experimental studies have been conducted on animal models, because it would be unethical to administer high doses of AASs over prolonged periods of time. Much remains to be investigated about the basic mechanisms in humans. Moreover, the habit of polydrug abuse makes it hardly possible to distinguish the toxic effects of AASs from those caused by other drugs [138]. In addition, a general limitation of human studies is the fact that data about the modality and doses of AAS use/abuse are often self-reported. Furthermore, there is a tendency to abuse multiple substances at the same time. Lastly, the susceptibility of individuals is influenced by genetic factors that are well known as key factors in developing adverse events [139].

In a systematic review of the literature on online resources, we found a total of 766 articles, but only 33 studies reported data about subjects abusing ND. Most reported adverse effects were endocrine (18 studies, 42%), cardiovascular (six studies, 14%), skin (five studies, 12%), and psychiatric (four studies, 9%) disorders. 

Side effects secondary to the use of ND may arise in some cases since the first administration. Some side effects regress quickly after suspension (for example, side effects on the skin or blood changes). However, there are some side effects that persist for some time and may not regress completely on suspension (for example, side effects on the reproductive, hormonal, nervous, and immune systems, organ damage to the kidney and liver, and cardiovascular or behavioral changes).

The result of this review highlights the need to investigate the consequences of the use of these substances because, currently, there are discordant results in many studies.

## Figures and Tables

**Table 1:** Classification of anabolic androgenic steroids (AAS).

Class	Chemical Structure	Examples
I	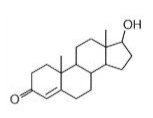	Testosterone propionate
II	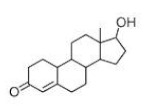	Nandrolone decanoate
III	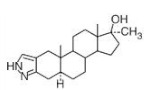	Stanozolol

**Table 2:** Demographic data.

	Population	Age (Years) ^1^
Males	346 (87%)	33.3
Females	63 (13%)	53.3
**Total**	409	34.8 (S.D. 12.8)
Treatment	313	
Non-treatment	96	

^1^ Age is considered only when both age and gender are reported. S.D.: standard deviation.

**Table 3:** Adverse effects.

	Studies	Common Reports
Endocrine	18	(virilization, gynecomastia, hormonal disorder, cholesterol and lipid, genital and infertility issues)
Cardiovascular	6	(vascular damage, coagulation disorders, arterial hypertension)
Skin	5	(pricking, acne, skin spots)
Psychiatric	4	(aggressiveness, mood disorders, sleep disorders, anxiety)
Musculoskeletal	2	(tendon ruptures)
Excretory	2	(organ damage)
Gastrointestinal	2	(organ damage, liver adenomas)
Neurological	1	(seizures)
Immune	1	(chronic infection relapse)
Respiratory	1	(sleep apnea syndrome)
Genetic	1	(genetic damage)

## Appendix A. General Data Obtained by Analyzing the Studies Included in This Literature Review

**Name**	**Authors**	**Publication Date**	**Males**	**Females**	**Age**	**Abuse**	**Adverse Effects**
Suspected reactivation of extrapulmonary tuberculosis focus after non-medical abuse of anabolic androgenic steroids: a case report	Singh V, Batta A.	2019	1	0	21	Yes	Immune system disorders
Side effects of anabolic steroids used by athletes at Unaizah Gyms, Saudi Arabia: a pilot study	Almaiman AA	2019	12	0	30	Yes	Kidney injuries, Skin disorders
Evaluation of anabolic steroid induced renal damage with sonography in bodybuilders	Kantarci UH	2018	22	0	25	Yes	Kidney injuries, Cardiovascular disorders
Anabolic steroid abuse: what shall it profit a man to gain muscle and suffer the loss of his brain?	Chowdhury P	2017	1	0	30	Yes	Cardiovascular disorders, Neurological disorders
Impact of single-dose nandrolone decanoate on gonadotropins, blood lipids and HMG CoA reductase in healthy men	Gårevik N, Börjesson A, Choong E, Ekström L, Lehtihet M.	2016	11	0	37,3	No	Endocrine disorders
Iatrogenic dependence of anabolic-androgenic steroid in an Indian non-athletic woman	Tripathi A, Tekkalaki B, Saxena S, Dandu H.	2014	1	0	55	Yes	Psychiatric disorders, Endocrine disorders, Skin disorders
Chromosome damage and cytotoxicity in oral mucosa cells after 2 months of exposure to anabolic steroids (decadurabolin and winstrol) in weight lifting	Martins RA, Gomes GA, Aguiar O Jr, Medalha CC, Ribeiro DA.	2010	15	0	Not available	Yes	Genetic disorders
Paraffinoma and ulcer of the external genitalia after self-injection of nandrolone	Balighi K, Farsinejad K, Naraghi ZS, Tamizifar B.	2008	1	0	56	Yes	Skin disorders
Acne induced by ‘Sus’ and ‘Deca’	Walker SL, Parry EJ.	2006	1	0	22	Yes	Skin disorders
Dilated cardiomyopathy and acute liver injury associated with combined use of ephedra, gamma-hydroxybutyrate, and anabolic steroids	Clark BM, Schofield RS.	2005	1	0	40	Yes	Kidney injuries, Cardiovascular disorders, Liver injuries, Endocrine disorders
Hepatocellular adenomas associated with anabolic androgenic steroid abuse in bodybuilders: a report of two cases and a review of the literature	Steensland P, Blakely G, Nyberg F, Fahlke C, Pohorecky LA.	2005	2	0	29	Yes	Liver injuries
Androgen-induced cerebral venous sinus thrombosis in a young body builder: case report	Kurling S, Kankaanp A, Ellermaa S, Karila T, Sepp T.	2004	1	0	22	Yes	Cardiovascular disorders
Effect of Nandrolone Decanoate on serum lipoprotein (a) and its isoforms in hemodialysis patients	Socas L, Zumbado M, PÃ©rez-Luzardo O, Ramos A, PÃ©rez C, HernÃ¡ndez JR, Boada LD.	2004	47	17	63	No	Cardiovascular disorders, Endocrine disorders
Effects of androgenic-anabolic steroids on apolipoproteins and lipoprotein (a)	Steensland P, Hallberg M, Kindlundh A, Fahlke C, Nyberg F.	2004	19	0	31	No	Endocrine disorders
Successful treatment of anabolic steroid-induced azoospermia with human chorionic gonadotropin and human menopausal gonadotropin	Menon DK	2003	1	0	37	Yes	Endocrine disorders
Measurement of androgen and estrogen receptors in breast tissue from subjects with anabolic steroid-dependent gynecomastia	Calzada L, Torres-Calleja J, Martinez JM, Pedron N.	2001	12	0	28	No	Endocrine disorders
Reversible hypogonadism and azoospermia as a result of anabolic-androgenic steroid use in a bodybuilder with personality disorder. A case report	Boyadjiev NP, Georgieva KN, Massaldjieva RI, Gueorguiev SI.	2000	1	0	20	Yes	Psychiatric disorders, Endocrine disorders
Androgens for the treatment of anemia in peritoneal dialysis patients	Navarro JF	1998	9	0	Not available	No	Endocrine disorders
A report on alterations to the speaking and singing voices of four women following hormonal therapy with virilizing agents	Baker J	1999	0	4	43	No	Endocrine disorders
Nandrolone decanoate is a good alternative for the treatment of anemia in elderly male patients on hemodialysis	Gascón A, Belvis JJ, Berisa F, Iglesias E, Estopiñán V, Teruel JL.	1999	14	0	Not available	No	Endocrine disorders
Effects of nandrolone decanoate (Decadurabolin) on serum Lp(a), lipids and lipoproteins in women with postmenopausal osteoporosis	Lippi G, Guidi G, Ruzzenente O, Braga V, Adami S.	1997	0	19	Not available	No	Endocrine disorders
Androgen therapy for anemia of chronic renal failure. Indications in the erythropoietin era	TeruelJL	1996	67	17	Not available	No	Endocrine disorders
Exogenous androgens influence body composition and regional body fat distribution in obese postmenopausal women—a clinical research center study	Lovejoy JC	1996	5	5	Not available	No	Psychiatric disorders, Endocrine disorders, Skin disorders
Bilateral rupture of the quadriceps tendon associated with anabolic steroids	Liow RY	1995	1	0	29	Yes	Musculoskeletal injuries
Assessment of attentional bias and mood in users and non-users of anabolic-androgenic steroids	Bond AJ	1995	32	0	23,2	Yes	Psychiatric disorders
An acute myocardial infarction occurring in an anabolic steroid user	Huie MJ	1994	1	0	Not available	Yes	Cardiovascular disorders
Virilization of the voice in post-menopausal women due to the anabolic steroid nandrolone decanoate (Decadurabolin). The effects of medication for one year	GerritsmaEJ	1994	0	0	Not available	No	Endocrine disorders
Rupture of the triceps tendon associated with steroid injections	Stannard JP	1993	1	0	35	Yes	Musculoskeletal injuries
Anabolicsteroids in postmenopausalosteoporosis	Need AG	1993	0	0	Not available	No	Endocrine disorders
Exuberant local tissue reaction to intramuscular injection of nandrolone decanoate (Decadurabolin)—a steroid compound in a sesame seed oil base—mimicking soft tissue malignant tumors: a case report and review of the literature	Khankhanian NK	1992	1	0	Not available	Yes	Connective disorders
Anabolic steroid-associated hypogonadism in male hemodialysis patients	Maeda Y	1989	66	0	Not available	No	Endocrine disorders
Regeneration of murine megakaryocytopoiesis and the hematopoietic inductive microenvironment after sublethal whole body irradiation by treatment with an anabolic steroid	Johnson MW	1984	0	1	54	No	Endocrine disorders, Sleep Apnea Syndrome
